# Obstetric outcomes following ovarian hyperstimulation syndrome in IVF – a comparison with uncomplicated fresh and frozen transfer cycles

**DOI:** 10.1186/s12884-022-04903-9

**Published:** 2022-07-18

**Authors:** Hadas Ganer Herman, Yossi Mizrachi, Eran Horowitz, Ariel Weissman, Ben Sabban, Ohad Gluck, Arieh Raziel, Michal Kovo

**Affiliations:** 1 In-Vitro Fertilization Unit, the Edith Wolfson Medical Center, Holon, affiliated with the Sackler Faculty of Medicine, Tel Aviv University, Tel Aviv, Israel; 2 Department of Obstetrics & Gynecology, the Edith Wolfson Medical Center, Holon, affiliated with the Sackler Faculty of Medicine, Tel Aviv University, Tel Aviv, Israel; 3 Department of Obstetrics and Gynecology, Meir Medical Center, Kfar Saba, affiliated with the Sackler Faculty of Medicine, Tel Aviv University, Tel Aviv, Israel

**Keywords:** In Vitro Fertilization (IVF), Ovarian Hyperstimulation Syndrome (OHSS), Fresh embryo transfer, Frozen embryo transfer, Placental abruption

## Abstract

**Background:**

We aimed to assess the correlation between ovarian hyperstimulation syndrome (OHSS) in the early course of in vitro fertilization (IVF) pregnancies and obstetric outcomes.

**Methods:**

We identified records of patients admitted due to OHSS following IVF treatment at our institution between 2008 and 2020. Cases were included if pregnancy resulted in a live singleton delivery (OHSS group). OHSS cases were matched at a 1:5:5 ratio with live singleton deliveries following IVF with fresh embryo transfer (fresh transfer group) and frozen embryo transfer (FET group), according to maternal age and parity. Computerized files were reviewed, and maternal, obstetric and neonatal outcomes compared.

**Results:**

Overall, 44 OHSS cases were matched with 220 fresh transfer and 220 FET pregnancies. Patient demographics were similar between the groups, including body mass index, smoking and comorbidities. Gestational age at delivery, the rate of preterm births, preeclampsia and cesarean delivery were similar between the groups. Placental abruption occurred in 6.8% of OHSS pregnancies, 1.4% of fresh transfer pregnancies and 0.9% of FET pregnancies (*p*=0.02). On post-hoc analysis, the rate of placental abruption was significantly higher in OHSS pregnancies, compared with the two other groups, and this maintained significance after adjustment for confounders. Birthweights were 3017 ± 483, 3057 ± 545 and 3213 ± 542 grams in the OHSS, fresh transfer and FET groups, respectively (*p*=0.004), although the rate of small for gestational age neonates was similar between the groups.

**Conclusions:**

OHSS in the early course of IVF pregnancies is associated with an increased risk of placental abruption.

## Background

Ovarian hyperstimulation syndrome (OHSS) is a complication of controlled ovarian stimulation. Presentation varies, although in its severe form, this iatrogenic complication is potentially life threatening. Different strategies for reducing the risk of OHSS have been implemented, resulting in a decrease in incidence over the past decades. Current estimations are of an incidence of 5.3 for 1000 in vitro fertilization (IVF) cycles [1]. Despite effective measures aimed at reducing the occurrence of OHSS, this complication is still encountered in fertility treatments. As OHSS patients are generally younger and exhibit a favorable response to treatment, patients commonly conceive if transfer is performed, as thus pregnancy course and obstetric outcomes in such cases are of interest.

The pathogenesis of OHSS involves high estradiol levels and hCG triggering, resulting in a change to the cytokine/inflammatory milieu, including altered levels of vascular endothelial growth factor, angiotensin and interleukins. Subsequently, increased capillary permeability and blood volume reduction develop, with potential electrolyte imbalance and thrombosis. Both the high estradiol levels during stimulation, inflammatory response and altered vascular supply in OHSS could potentially affect implantation and placentation. Therefore, several previous studies have explored the course of pregnancies following OHSS, focusing on obstetric and perinatal outcomes. OHSS was found associated with low birthweight and preterm deliveries [1–6], while others found an association with shorter gestation, gestational diabetes mellitus and neonatal complications [7]. Two additional studies did not demonstrate these adverse outcomes following severe OHSS [8, 9].

All aforementioned studies employed a control group of IVF pregnancies. However, not all matched adequately for age, parity and importantly – included only stimulated cycles without a comparison to a group without stimulation, such as frozen embryo transfer (FET). Thus, we sought to investigate a cohort of patients hospitalized for OHSS, and compare them to IVF patients, following both stimulated cycles (fresh transfer) without OHSS and non-stimulated cycles (FET).

## Methods

This was a historic cohort study in a single university affiliated medical center. Severe OHSS was defined according to the presence of clinical and laboratory findings [10]. Clinical symptoms and signs included: intractable nausea or vomiting, severe abdominal pain, rapid weight gain (>one kilogram per day), clinical ascites, hydrothorax, pericardial effusion, severe dyspnea, oliguria or anuria and thromboembolism. Laboratory signs included: hematocrit > 55%, white blood cells > 25000 mL, creatinine > 1.6 mg/dL, Na< 135 mEq/L, K> 5 mEq/L or elevated liver enzymes. Hospitalization of all cases of severe OHSS is undertaken at our institution, as for select moderate cases unresponsive to ambulatory treatment, defined by sonographic ascites, hematocrit > 41% and white blood cells > 15000 mL [10]. We identified the medical records of women hospitalized for OHSS between 2008 and 2020, according to ICD9 coding. We subsequently excluded cases in which patients did not conceive following hospitalization, cases of OHSS following ovulation induction (not IVF) and cases with multiple pregnancy. All cases of OHSS following IVF, which ended in a live singleton delivery at 24 weeks gestation or more were included in the study group. We also contacted patients for whom a pregnancy test was not performed during hospitalization, or for which a positive pregnancy result was available but did not deliver at our institution, to ask regarding pregnancy course. Women were asked if they conceived following treatment, and if so, did the pregnancy result in a live singleton birth at term. If so, these cases were also included in the study group, provided full information from the woman regarding pregnancy and delivery course, and neonatal weight.

The study group was matched at 1:5:5 ratio with IVF pregnancies following fresh and FET cycles, that were not complicated by OHSS, and resulted in live singleton deliveries. Matching was performed according to parity and maternal age (± one year). We excluded pregnancies for which data was missing regarding mode of conception or those with no prenatal care.

Chronic hypertension, gestational hypertension and preeclampsia, were defined according to the American College of Obstetrics and Gynecology [11], and pregestational and gestational diabetes mellitus according to the American Diabetes Association [12]. Preterm birth was considered a delivery prior to 37 weeks gestation. Small for gestational age was defined as birthweight below the 10^th^ percentile of the local population-based nomograms [13]. Placental abruption was defined as per ICD9 coding by physician during labor. Abruption was coded as based on physician’s clinical impression, and histopathological placental examination was performed in all suspected cases to affirm diagnosis as per department protocol.

The study was approved by the Edith Wolfson Medical Center Ethical Committee on January 1^st^, 2020, institutional review board #0183-19-WOMC. All methods were carried out in accordance with relevant guidelines and regulations.

Data analysis was performed using SPSS software v25 (IBM, USA). Continuous variables were compared by Anova test and categorical variables were compared by the chi square test of fisher’s exact test, as appropriate. A post hoc T-test analysis was performed in cases of statistical significance, to demonstrate which inter-group differences contributed to significance. All tests were two tailed, and the threshold for statistical significance was defined as p-value <0.05.

## Results

A total of 182 patients were admitted to the hospital with OHSS during the study period, of whom 80 subsequently delivered at our institution. Of these deliveries, 31 were eligible for inclusion, as the remainder were multiple gestations and two cases of terminations of pregnancy. The remaining 102 files were reviewed, and patients contacted if data was not available by computer – 13 women had a singleton live delivery at another institution, reported all outcomes of interest, and were included in the OHSS group. Among the remaining 89 patients - in five, embryo transfer was not performed due to OHSS, 29 patients did not conceive as a result of treatment, 14 conceived and had a miscarriage and one intrauterine fetal demise, 8 delivered twins, two underwent termination of pregnancy due to fetal malformations and one woman was in her third trimester and had yet to deliver. Data was unavailable for 29 patients. Thus, the OHSS group consisted of 44 pregnancies, matched to 220 fresh transfer pregnancies and 220 FET pregnancies. Among FET pregnancies – 129 were following programmed hormonal-based transfer, 65 were modified natural cycles and for 26 data regarding FET was unavailable.

Demographic characteristics of the OHSS, fresh transfer and frozen transfer groups are presented in Table 1. As per matching, no differences were noted in patient age and nulliparity rate between the groups. The average BMI and rate of smoking, chronic hypertension and pregestational diabetes mellitus were also similar between the groups. A total of five cases of Mullerian abnormalities were noted in the cohort (non-significant between the groups) – two cases of uterine didelphys, two cases of unicornuate uterus and one case of a bicornuate uterus.

**Table 1:** Demographic characteristics of the OHSS, fresh transfer and frozen transfer groups

	OHSS group *n*=44	Fresh transfer group *n*=220	Frozen transfer group *n*=220	P
Age, years	31.2 ± 5.1	31.2 ± 5.1	31.3 ± 5.1	0.99
BMI, kg/m^2^	24.2 ± 3.4	24.8 ± 5.9	24.4 ± 5.2	0.78
Nulliparous	26 (59.1%)	130 (59.1%)	130 (59.1%)	>0.99
Smoking	7 (15.9%)	27 (12.2%)	21 (9.5%)	0.40
Chronic hypertension	0	4 (1.8%)	2 (0.9%)	0.50
Pregestational diabetes	0	8 (3.6%)	5 (2.2%)	0.34

Data presented as mean ± SD or n (%) as appropriate

*SD* standard deviation, *n* Number, *BMI* body mass index, pre-gestational, *DM* diabetes mellitus

Pregnancies following OHSS, fresh transfer and FET were characterized by a similar gestational age at delivery and a similar rate of preterm delivery (Table 2). No differences were detected in the rate of preeclampsia or gestational hypertension, gestational diabetes mellitus and placenta previa. Placental abruption occurred in 6.8%, 1.4% and 0.9% of deliveries in the OHSS, fresh transfer and FET groups, respectively (p=0.02), while in a post-hoc analysis this rate was significantly different between OHSS pregnancies and the two other groups. Birthweight was also significantly different between the groups – 3017 ± 483 grams in the OHSS group, 3057 ± 545 grams in the fresh transfer group and 3213 ± 542 grams in the FET group (p=0.004). In post hoc analysis this difference was significant between the FET and two other study groups.

**Table 2:** Obstetric and neonatal outcome of the OHSS, fresh transfer and frozen transfer groups

	OHSS group *n*=44	Fresh transfer group *n*=220	Frozen transfer group *n*=220	P
Gestational age at delivery, weeks	38.6 ± 1.9	38.7 ± 2.0	38.9 ± 1.7	0.43
Preterm delivery	6 (13.6%)	17 (7.7%)	18 (8.2%)	0.42
Preeclampsia	1 (2.3%)	10 (4.5%)	9 (4.1%)	0.78
Gestational hypertension	0	3 (1.4%)	8 (3.6%)	0.15
Gestational diabetes	3 (6.8%)	11 (5.0%)	22 (10.0%)	0.13
Placenta previa	0	4 (1.8%)	2 (0.9%)	0.50
Placental abruption	3 (6.8%)^a,b^	3 (1.4%)^a^	2 (0.9%)^b^	**0.02**
Instrumental delivery	3 (6.8%)	15 (6.8%)	20 (9.1%)	0.65
Cesarean delivery	10 (22.7%)	47 (21.4%)	66 (30.0%)	0.10
Blood transfusion, ante / postpartum	1 (2.3%)	3 (1.4%)	8 (3.6%)	0.30
Birth weight, grams	3017 ± 483^a^	3057 ± 545^b^	3213 ± 542^a,b^	**0.004**
Small for gestational age	5 (11.3%)	30 (13.6%)	17 (7.7%)	0.13

Data presented as mean ± SD or n (%) as appropriate.

*SD* standard deviation, *n* number, Premature delivery – under 37 weeks

A logistic regression model was composed, in which placental abruption served as the dependent variable, while OHSS, gestational age and smoking served as independent variables (Table 3). OHSS was found independently associated with placental abruption [OR 6.31, 95%CI 1.44-27.56, *p*=0.01].

**Table 3:** Logistic regression model for placental abruption

	OR	95% C.I		P
		Lower	Upper	
OHSS	6.31	1.44	27.56	0.01
Gestational age	0.83	0.65	1.07	0.15
Smoking	1.01	0.11	8.65	0.98

*OR* odds ratio*, C.I* confidence interval*. OHSS* ovarian hyperstimulation syndrome

A sub analysis was performed, in which FET cases were analyzed separately for programmed cycles (*n*=129) and natural modified cycles (n=65) and excluded 26 cases in which FET cycle type was unknown. Analysis is hereby described and not presented in a table. Demographic characteristics were similar between the group, while birthweight was significant between the groups – 3238 ± 512 grams for natural modified cycles, 3188 ± 562 grams for programmed cycles, 3057 ± 545 grams for fresh cycles and 3017 ± 483 grams for OHSS cases, p=0.02. On post hoc analysis, significance was between natural modified cycles and fresh cycles (p=0.01), natural modified cycles and OHSS cases (*p*=0.02) and between programmed cycles and fresh cycles (*p*=0.03). The rate of placental abruption was also significant, 3.0% in natural modified cycles, 0% in programmed cycles, 1.3% in fresh cycles and 6.8% in OHSS cases, *p*=0.01. On post hoc analysis, this was significant between programmed cycles and OHSS cases (*p*=0.01) with a trend for significance between fresh cycles and OHSS cases (*p*=0.06).

## Discussion

The objective of our study was to assess obstetric outcomes in pregnancies complicated by OHSS in early course following IVF. We compared OHSS cases to controls following both fresh and FET IVF cycles, and demonstrated a lower birthweight with OHSS and uncomplicated fresh transfers as compared to FET, and a significantly higher risk of placental abruption following OHSS, as compared to both fresh and FET cycles. This increased risk was independent of gestational age and smoking.

Controlled ovarian hyperstimulation is associated with an increase in estradiol levels, correlated to the amount and size of developing follicles. While the recruitment of more follicles generally increases patients’ chances of conception, the often supraphysiological levels of estradiol may affect implantation and subsequent placentation following transfer. Past in vivo studies have studied the effect of assisted reproductive procedures on placental development, and found overgrowth of the placental junctional zone with superovulation [14]. Superovulation has also been associated with altered expression of significant genes involved in in endometrial remodeling during early implantation [15]. These pathogeneses may explain the adverse correlation between birthweight and peak estradiol serum levels repeatedly demonstrated in past studies [16–22]. Furthermore, high estradiol levels have been linked to additional placental related adverse outcomes, including hypertensive disorders of pregnancy, premature preterm rupture of membranes and placenta previa [21, 22]. Although OHSS represents an “endpoint” to the continuum of peak serum estradiol levels attained following stimulation, this is probably not the sole pathogenesis of adverse pregnancy outcomes associated with it. OHSS entails a hypercoagulable state, and is associated with a systemic inflammatory response, and altered cytokine milieu [23, 24], which may in turn negatively affect the embryo-endometrial interaction.

Several past studies have explored the course of pregnancies complicated by OHSS following IVF, but few have focused on obstetric outcomes in singleton pregnancies. Hu et al compared patients hospitalized for moderate to severe OHSS to controls matched by age, ovarian reserve markers and multiple gestations [7]. They noted earlier gestational age at delivery, a higher rate of gestational diabetes mellitus and more neonatal complications with OHSS. Notably, almost half of pregnancies were multiple gestations, with no sub analysis for singletons, and information was not provided for zygosity and parity, which may affect outcomes. Haas and colleagues compared severe OHSS pregnancies following IVF or ovulation induction with IVF controls, matched by age and infertility etiology [2]. They found that patients with singletons following severe OHSS delivered at an earlier gestational age and had smaller babies, but multiple gestations were not at increased risk following OHSS. Three additional past studies have noted a higher rate of low birthweight, hypertensive disorders of pregnancy, gestational diabetes and placental abruption following OHSS [4–6], while two other studies did not demonstrate any differences from matched controls [8, 9].

The major finding of our study, in line with previous reports [5], is an increased rate of placental abruption following severe OHSS, as confirmed by logistic regression analysis. We demonstrated an incidence of 6.8% in the OHSS group, a significantly higher rate than in uncomplicated IVF pregnancies and following FET, and significantly higher than the previously reported 1% for all deliveries at our institution [25]. This finding is of utmost importance since placental abruption is one of the gravest complications of pregnancy with both maternal and neonatal consequences. It should be further explored whether this group of patients can benefit from closer pregnancy surveillance and/or from therapeutic interventions that may help reduce the risk. Of interest is that we did not demonstrate a difference in additional placental related complications, mainly birthweight following OHSS and fresh transfers, although this may relate to the overall low number of severe OHSS cases in our cohort. We did confirm a lower birth weight with stimulated IVF cycles as compared to FET, as previously demonstrated [26–28].

Study limitations refer first and foremost to the size of the study groups. The number of OHSS cases at our institution was relatively high for this now rare complication, most probably in light of the high rate of IVF cycles in our country (due to complete state funding) and higher number of fresh transfers earlier in the cohort period when freezing techniques were poor. Yet, we chose to include only singleton live births following IVF in our analysis, which limited the number of cases analyzed. A larger number of cases would have been powered for smaller differences in outcomes, such as in birthweight. Thus, a lack of power calculation must be taken into account when interpreting the results, as sample size was based on maximal sample obtained. We also assumed that programmed FET cycles entail a low level of serum estradiol, and thus included both programmed and natural FETs as controls. A larger number of cases may have allowed for a more suitably powered sub analysis of natural and programed FET cycles, to better account for the different obstetric outcomes associated with the two in past studies [29]. Second, as not all IVF cycles were performed at our institution, certain variables of interest were missing for analysis, such as infertility etiology, peak estradiol level during stimulation and number of embryos transferred. In a prospective study design, all patient data from IVF cycles would be available for analysis, and a uniform protocol for the screening of patients for OHSS would be applied (such as according to number of oocytes retrieved, peak estradiol levels, etc).

Study strengths refer to its design, which included matched groups of controls of both fresh and FET IVF pregnancies. The study also uniquely focuses solely on singleton IVF pregnancies, and accounts for age and parity for controls, both significant influencers of obstetric outcomes.

## Conclusions

In conclusion, IVF pregnancies following OHSS in early pregnancy course entail a higher risk of placental abruption, while birthweight is comparable to uncomplicated IVF pregnancies, but lower than FET ones. This finding is in line with previous studies pointing to a correlation between serum estradiol levels and placental complications. Despite the decreasing incidence of OHSS in recent years due to improved freezing and triggering techniques, the findings of our study could be extrapolated to non-complicated stimulated cycles, while considering the adverse relation between estradiol and obstetric complications.

## Data Availability

The datasets generated and/or analysed during the current study are not publicly available due to institutional regulations, but are available from the corresponding author on reasonable request.

## Abbreviations

OHSS: Ovarian Hyperstimulation Syndrome
IVF: In Vitro Fertilization
FET: Frozen embryo transfer
